# Dr. Lee’s Article

**Published:** 1891-11

**Authors:** 


REFERRING to Dr. Lee's article in this issue: By oversight the explanation of the cuts was omitted. The first cut represents the introduction of the bone rings in circular enterrorraphy; No. 2 is the Senn decalcified bone-plate (medium) actual size; No. 3 shows four rings from transverse section of the femur of an ox—slightly reduced in size—prepared and kept for use.

